# Intraoperative Three-Dimensional Imaging in Selective Decompression for Lumbar Spinal Stenosis: A Useful Tool in Theory but Also in Everyday Practice?

**DOI:** 10.1155/2011/108438

**Published:** 2011-05-23

**Authors:** Uwe Max Mauer, Ulrich Kunz, Chris Schulz

**Affiliations:** Department of Neurosurgery, German Armed Forces Hospital of Ulm, Oberer Eselsberg 40, 89070 Ulm, Germany

## Abstract

*Background.* We conducted a pilot study to investigate the value of an Iso-C3D imaging system in determining the extent of decompression of lumbar spinal stenosis during surgery. We now address the question whether this imaging has become a routine tool. *Material and Methods.* Ten patients who underwent unilateral decompression for lumbar spinal stenosis were intraoperatively examined using the Iso-C3D imaging system. Four years after this study, we investigated whether this intraoperative imaging modality is still being used. *Results.* Evaluable images were intraoperatively obtained for all patients. In two cases, the surgical procedure was changed on the basis of the images. Myelography did not provide any additional information. In the four years following the study, this intraoperative imaging technique has not been used again. *Conclusion.* Intraoperative imaging using the Iso-C3D system provides additional safety. It, however, has not become established as a routine procedure.

## 1. Introduction

Lumbar spinal stenosis is a degenerative disease of the lumbar spine and typically presents with symptoms that are mainly caused by spondylosis and hypertrophy of the ligamentum flavum. Spondylosis in association with disc generation can additionally cause relative instability. Over recent years, minimally invasive procedures have been increasingly used in the treatment of degenerative lumbar spinal stenosis. Before the use of minimally invasive procedures, laminectomy was the treatment of choice but is now performed only in rare cases. Laminectomy is associated with an iatrogenic increase in the existing instability and in many cases with an insufficient decompressive effect especially laterally [1]. For this reason, bilateral selective decompression or, in other words, interlaminar fenestration with the removal of as much bone tissue as necessary is widely used. Some hospitals advocate a unilateral procedure that crosses the midline in order to decompress the contralateral side as well [2]. Unilateral selective decompression involves undercutting vertebral arches with a view to removing thickened portions of ligamentum flavum and spondylophytes until the contralateral foramen is reached. Direct intraoperative imaging has thus far been performed using intraoperative computed tomography (CT) or magnetic resonance imaging (MRI), which both, however, require time as well as material and financial resources. Moreover, intraoperative monitoring using MRI involves substantial costs. Since its introduction, the Iso-C3D imaging system (Siemens, Erlangen, and Germany) has offered a relatively simple and time-effective intraoperative diagnostic imaging tool that, similar to CT, allows a patient to be studied without requiring patient repositioning on the operating table. Our objective was therefore to investigate whether this imaging system allows us to assess the effectiveness of unilateral decompression during surgery. 

Four years ago, we formulated and investigated two hypotheses. Firstly, intraoperative imaging with an Iso-C3D system can demonstrate the extent of decompression of neural structures on the contralateral side. Secondly, intrathecal contrast agent administration increases the effectiveness of imaging with an Iso-C3D system in terms of the first hypothesis. We found that the Iso-C3D system was a generally useful and effective imaging tool and published these results [3]. 

Following a brief description of our previous study, we address the question as to whether intraoperative 3D imaging has become a routine tool in everyday clinical practice.

## 2. Material and Methods

In our earlier study, 10 patients (4 women and 6 men) with a mean age of 67 years (median age: 63 years, range: 55–79 years) underwent intraoperative imaging with an Iso-C3D system following unilateral selective decompression for spinal stenosis. In 5 patients, intraoperative imaging was performed without contrast enhancement. Five patients underwent intraoperative imaging with myelography, which involved the direct injection of contrast material into the dural sac. Following coaxial alignment at the level of surgery, 100 single images were acquired and 256 reconstructions were obtained for every patient. The patients were placed on a carbon fiber table and ventilated with 100% oxygen for 5 minutes before scanning. They were not ventilated during scanning. All metal objects were removed from the operative site before scanning. Wound cavities were filled with a physiological saline solution. After the scans were acquired, reconstructions were obtained in the three standard planes parallel to the disk spaces and the sagittal axis of the spine. The reconstructed images were evaluated together with the surgeon and compared with preoperative CT and MRI scans. Every patient had undergone preoperative MRI of the lumbar spine and 9 of the 10 patients had also undergone CT of the lumbar spine. 

Four years after the study, we examined hospital records in order to determine how often the method described above has since been used.

## 3. Results

**Figure 1:**
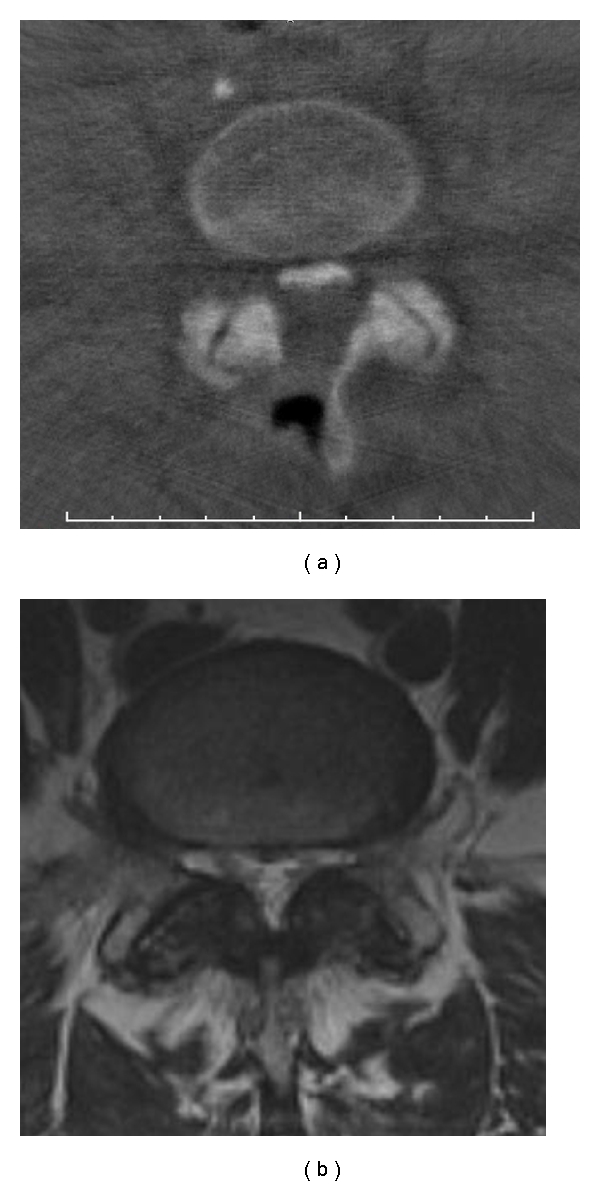
(a) Axial reconstruction demonstrating decompression from the left side with sufficient decompression on the right side. (b) Preoperative MR image of the same patient as in (a).

**Figure 2:**
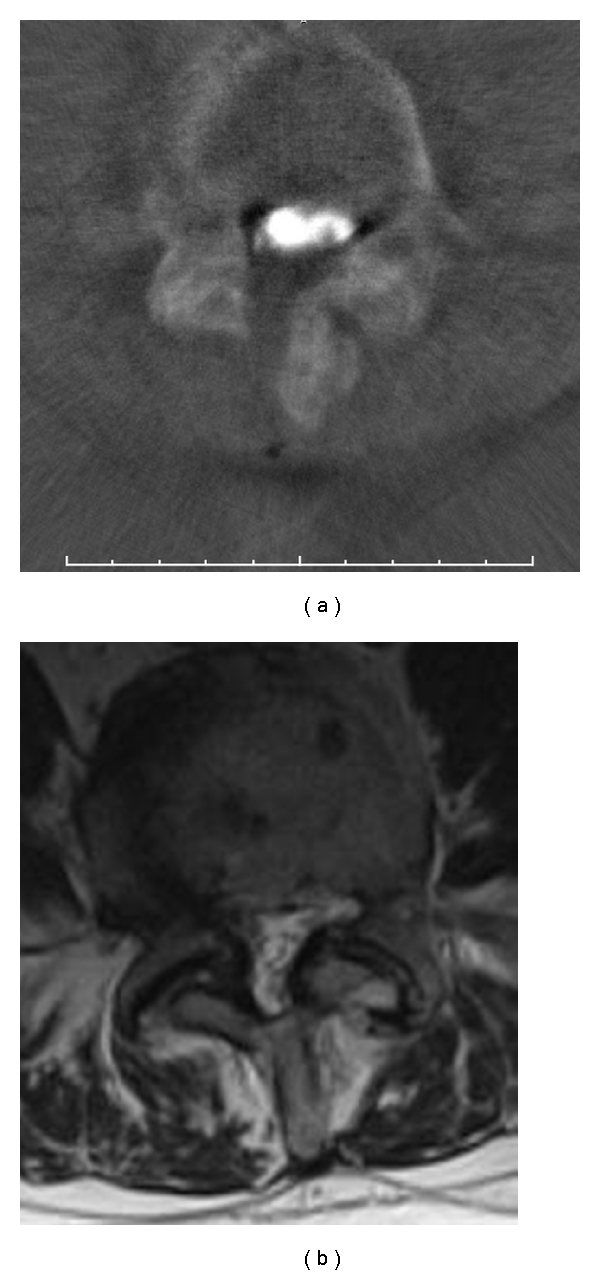
(a) Axial reconstruction with myelography demonstrating decompression from the left side with intraoperative myelography and insufficient decompression of the recess on the right side. (b) Preoperative MR image of the same patient as in (a).

Our study from 2006 produced the following results. We obtained evaluable scans for all patients in the first attempt. The quality of the images allowed us to assess the extent of decompression on the contralateral side in every case (Figures 1(a) and 1(b)). Especially when compared with preoperative MR images, the intraoperative non-contrast-enhanced three-dimensional reconstructions clearly revealed the extent of decompression. In two cases, we changed the surgical procedure on the basis of the scans in order to further decompress the contralateral side (Figures 2(a) and 2(b)). The duration of surgery increased by not more than 20 minutes in both cases. No patient developed an infection or had an allergic reaction to the contrast material. All patients had normal preoperative thyroid levels and no patient developed hyperthyroidism. Likewise, all patients had normal preoperative renal function. No accumulation or leakage of cerebrospinal fluid was observed in any of the patients who underwent myelography. One patient exhibited a minor pressure-induced lesion in the face.

The main problem was to mix the contrast material with cerebrospinal fluid (CSF) during myelography. In a normal radiological examination, the patient can be raised or lowered as required. This, however, was possible only to a limited extent in the surgical setting. Myelography thus provided no important additional information. 

Our findings thus fully confirmed our first hypothesis and refuted the second hypothesis. 

At the end of the study period and at least over a period of one year after surgery, all 10 patients were found to have greatly benefited from the surgical procedure. Nine patients remained fully satisfied and reported considerable pain relief and neurological improvement when compared to their preoperative condition. One patient with further spinal canal compression at two levels above the site of surgery complained again of symptoms, which, however, have not yet required surgery. 

Since the end of the study, no patient has been examined intraoperatively using the technique described above. The Iso-C3D imaging system has regularly been used during the insertion of a spinal internal fixation device to monitor the position of the implant, during anterior fixation of fractures of the odontoid process and during posterior fusion of the atlas and axis but it has since never been used for monitoring selective decompression for spinal stenosis.

## 4. Discussion

Unilateral decompression is a generally accepted method and has been described in several publications [2, 4]. Assessing the extent of decompression of the contralateral side, however, appears to be a problem. Intraoperative Iso-C3D imaging allowed us to visualize the extent of decompression during the surgical procedure. As the contrast material flows ventrally and does not properly mix with CSF, myelography is unlikely to provide important additional information. The intraoperative use of myelography together with the Iso-C3D imaging system has not yet been addressed. Since the publication of our study [3] in 2006, only one paper on the assessment of lumbar spinal decompression [5] and one paper on the evaluation of decompression of cervical spinal canal stenosis [6] have been published. During the same period, a much larger number of studies addressed the intraoperative monitoring of the placement of pedicle screws [7–9] and other implants used for the fixation of fractures of the extremities [10]. The aforementioned studies were performed with a Siemens Iso-C3D imaging system. A Ziehm Vario 3D system, however, can be used in a similar way [1]. 

Patel et al. [5] also performed intraoperative myelography in their study. Unlike us, however, they found that this examination method provided additional useful diagnostic information and noticed a marked learning curve. In their opinion, this method is particularly suitable for minimally invasive cases where the usual clinical intraoperative assessment is unavailable and also in unusual and complex cases. They did not suggest that myelography should be performed in routine clinical practice. 

Baldauf et al. [6] conducted a similar study and examined patients with cervical spinal canal stenosis. In 3 of 25 cases, intraoperative imaging showed that their clinical evaluation of the extent of decompression had been inadequate and that further surgery was required. Like many other authors, they, too, monitored the position of implants. Although they concluded that the method is useful and helps avoid surgical revisions, they did not advocate the routine use of this technique. 

Compared with intraoperative MRI, imaging with an Iso-C3D system requires far less time and fewer resources and incurs far lower costs. Hüfner et al. [11] calculated that a rate of avoided revisions of at least 5% is sufficient to justify the purchase and use of an intraoperative 3D imaging system. For their cost calculation, they used the following formula: (annual fixed costs) + (costs per scan × number of cases) − (revision costs × revision rate × number of cases).

This imaging modality is, however, associated with a moderately higher level of radiation exposure than standard spiral CT of the lumbar spine over a length of 10 cm [12] and with a five-fold higher level of exposure to scattered radiation. For this reason, an examiner should maintain a distance of 3.5 meters from the radiation source [4]. The procedure itself is safe. 

Despite the aforementioned advantages, this method has not become a routine tool in everyday clinical practice. There are several reasons for this. For selective decompression in the region of the lumbar spinal canal, it is more appropriate to place patients in the knee-elbow position than to place them in the prone position and use cushions for patient support. An intraoperative scan, however, can only be performed with the patient in the prone position and not in the knee-elbow position. For this reason, surgeons must decide on a case-by-case basis whether the use of intraoperative imaging justifies the additional time required to reposition the patient and to cover the surgical site again with sterile drapes in routine clinical procedures. 

Although the method presented here is more affected by artifacts from metal implants than standard computerized tomography [11], it has become a routine tool for monitoring the position of implants in everyday clinical practice in many institutions [7–9]. Implant malposition is usually a problem that is immediately obvious and requires surgical revision. By contrast, insufficient decompression is not necessarily of clinical relevance and is not as easily detectable as the presence of a screw in the spinal canal. We believe that this is the main reason why a surgeon is usually willing to utilize intraoperative imaging modalities in order to monitor the position of an implant but is reluctant to use it to assess the extent of decompression intraoperatively. 

Although its effectiveness is undeniable, the intraoperative examination method described here is most likely to be used only in special cases in which the contralateral side is difficult to evaluate, for example, for anatomical or technical reasons.
